# Effectiveness and safety of percutaneous vertebroplasty in the treatment of spinal metastatic tumor

**DOI:** 10.12669/pjms.333.12276

**Published:** 2017

**Authors:** Yilin Liu, Yuqiang Wang, Liang Zhao, Ruipeng Song, Hongyu Tan, Limin Wang

**Affiliations:** 1 Yilin Liu, Department of Orthopedics, The First Affiliated Hospital of Zhengzhou University, Zhengzhou450052, China; 2 Yuqiang Wang, Department of Orthopedics, The First Affiliated Hospital of Zhengzhou University, Zhengzhou450052, China; 3 Liang Zhao, Department of Orthopedics, The First Affiliated Hospital of Zhengzhou University, Zhengzhou450052, China; 4 Ruipeng Song, Department of Orthopedics, The First Affiliated Hospital of Zhengzhou University, Zhengzhou450052, China; 5 Hongyu Tan, Department of Orthopedics, The First Affiliated Hospital of Zhengzhou University, Zhengzhou450052, China; 6 Limin Wang, Department of Orthopedics, The First Affiliated Hospital of Zhengzhou University, Zhengzhou450052, China

**Keywords:** Spinal metastatic tumors, Percutaneous vertebroplasty, Safety

## Abstract

**Objective:**

To evaluate the effectiveness, safety and feasibility of percutaneous vertebroplasty in the treatment of spinal metastatic tumor.

**Methods:**

Thirty-four patients with spinal metastatic tumor who received treatment in the First Affiliated Hospital of Zhengzhou University from May 2014 to June 2015 were selected. Totally fifty diseased vertebrae were treated by percutaneous vertebroplasty. The curative effects were evaluated according to visual analogue scale (VAS) score, Oswestry Dability Index (ODI) and dose of pain reliever. The leakage conditions of bone cement and clinical complications were observed. The patients were followed up for 3 to 12 months.

**Results:**

The average VAS score and ODI 24 h after treatment were much lower than those before treatment, and the difference had statistical significance (P<0.05). The average VAS score and ODI at different follow-up periods after treatment were not significantly different (P>0.05). During follow up, nine patients stopped taking pain reliever, the dose of 18 patients had obvious reduction, and 7 patients kept previous dose; the incidence of bone cement leakage was 38.25%. Six patients had fever after surgery, but recovered after expectant treatment; 2 patients felt uncomfortable in the right lower limbs, but relieved after expectant treatment.

**Conclusion:**

Percutaneous vertebroplasty can relieve pain efficiently, improve the daily living ability, and significantly enhance the living quality of patients with spinal metastatic tumors, with small trauma and high safety.

## INTRODUCTION

Bone metastasis is frequently seen in the spinal, and its incidence is 5% to 10% among tumor patients. Its incidence in thoracic vertebra is the highest (70%), followed by lumbar vertebra (20%) and cervical vertebra (10%). Breast carcinoma, pulmonary carcinoma and prostatic cancer are the most common primary lesions for patients with bone metastasis tumors.^1^,^2^ Patients with spinal metastatic tumors usually have clinical manifestations such as intense pain, neurological disorders and paralysis, which brings huge pains for patients and also severely affects the living quality and survival period of patients.^3^

The current treatment approaches for spinal metastatic tumors include chemotherapy, radiotherapy and surgery.^4^ Radiotherapy is the most common therapy; but it cannot improve neurological function, especially spinal paralysis, by relieving spinal instability induced by tumors. Moreover, radiotherapy is ineffective for preventing advanced collapse of patients with pathological fracture. As the indication range of open decompression surgery is narrow, most patients who have spinal metastatic tumor or vertebral hemangioma in more than two segments often go untreated; moreover, patients with advanced tumors are difficult to tolerate open decompression surgery due to heavy trauma and multiple complications. Percutaneous vertebroplasty, a kind of minimally invasive spinal treatment technology, has been applied in the treatment of intractable pain in recent years and has achieved good effects.^5^,^6^ This study aims at observing the clinical effects of percutaneous vertebroplasty in the treatment of spinal metastatic tumors and evaluate safety, thus to provide a reference for the clinical treatment.

## METHODS

Thirty-four patients who received percutaneous vertebroplasty in the hospital from May 2014 to July 2015 were retrospectively analyzed. Totally fifty vertebrae were included, including 3 cervical vertebrae, 24 thoracic vertebrae and 4 sacral vertebrae. There were 23 males and 11 females, aged from 38 to 74 years old. There were 16 cases of liver carcinoma, 7 cases of pulmonary carcinoma, 4 cases of breast cancer, 5 cases of colorectal carcinoma and 2 cases of gastric carcinoma. The tumors of all patients have been pathologically diagnosed. Before surgery, the patients underwent thoracolumbar spine computed tomography (CT) or Magnetic Resonance Imaging (MRI); 20 patients were diagnosed as pathological fracture in vertebrae, 15 patients had bone cortex rupture at vertebral posterior border, and 5 patients had nerve root compression, among which, 2 patients had weakened limbs muscle force. All patients had intense pain induced by spinal metastatic tumors in different sites. Twenty-four patients orally took opium analgesics and 10 patients orally took nonopioids; their Visual Analogue Scale (VAS) scores became larger than 4 points after medication. The study has been approved by the ethics committee of the hospital. The patients have signed informed consent. Those who had surgical contraindication, mental diseases, or cardiac, hepatic and liver function abnormality were excluded.

### Instruments and equipment

11 G or 13 G Bone puncture needle (Shanghai Kinetic Medical Co. Ltd., China), bone guider (KMC, USA), bone cement (Tecres S. P. A. Company, Italy) and cardiovascular imaging system (GE medical Systems SCS, Germany) were used.

### Preoperative preparation

All the cases were discussed in the clinical departments before surgery. Appropriate operation plans were designed based on the clinical manifestations, injured segments and imaging characteristics. The doctors were invited from relevant departs for consultation to exclude patients with surgical contraindications. Thorough countermeasures were formulated for the difficulties and risks that might be encountered during operation.

### The procedures of interventional operation

The first step was the insertion of bone puncture needle. Patients took a prone position on a Digital Subtraction Angiography (DSA) table (cervical vertebra took a supine position). The diseased vertebrae and puncture point were determined under the guidance of DSA. Then the puncture channels were narcotized one by one. The puncture needle was inserted into the center of the vertebrae along the puncture paths simulated before surgery. After the confirmation by adem position perspective, the needle core was removed. Then a kirschner wire was inserted and the outer sleeve of the bone puncture needle was pulled out. The bone guider was inserted to the position which was 1/3 before vertebrae along the kirschner wire. The second step was the infusion of bone cement. Bone cement was infused into a bone cement cannula (1.5 ml). The cannula was pushed into the diseased vertebrae under the real-time monitoring when the bone cement became viscous. The injection stopped when the bone cement closed to the vertebra posterior border or severe complications happened. Antibiotics were used one hour before surgery and one day after surgery to prevent infection.

### Observation indexes and evaluation criteria

**Fig.1:**
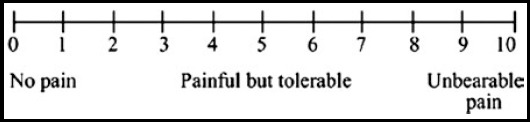
Diagram of VAS.
(2) Owestry Disability Index (ODI) was used to evaluate the degree of daily living disorder of the patients.^8^ The patients were followed up in the form of questionnaire. The scores were calculated after the patients filled up the scale. The ODI could be obtained by dividing the score by 50.
(3) The dose of pain reliever before and after surgery were compared to evaluate the pain relief.
(4) The leakage of bone cement and the incidence of complications were observed after surgery to measure the safety and reliability of the surgery.

(1) *VAS*: a direct line which was 10 cm long was drawn. 0 point stands for no pain and 10 points stands for the most intense pain.^7^ The patients were asked to point out the score according to their subjective pains. The distance from 0 to the corresponding point was regarded as VAS score. The diagram for VAS is shown in Fig.1.

## RESULTS

### Surgical results

**Fig.2:**
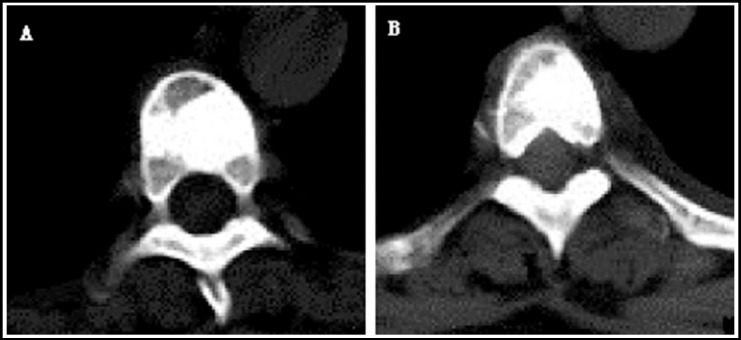
Postoperative CT scanning demonstrated good distribution of bone cement in T4 (A) and T7 (B) and pains relieved.

All patients completed percutaneous vertebroplasty under the guidance of DSA, and the success rate of surgery was 100%. The infusion amount of bone cement for each vertebra was 1~5 ml (average 3.22±0.84 ml), as shown in Fig.2. Of the 50 vertebrae, bone cement leaked out in 19 vertebrae (38.2%). The leakage of bone cement happened in paravertebral soft tissues (6 thoracic vertebrae and 4 lumbar vertebra), paravertebral veins (4 lumbar vertebrae), 4 intervertebral discs (2 thoracic vertebrae and 2 lumbar vertebrae) and canalis vertebralis (2 lumbar vertebrae). The leakage could be discovered under perspective during operation, and the infusion stopped immediately after leakage was observed.

### Comparison of VAS score and ODI values before surgery and at different follow-up periods after surgery

The average VAS score and ODI values 24 h after surgery were much lower than those before surgery, and the differences had statistical significance (P<0.05). The average VAS score and ODI values at different follow-up periods after surgery had no significant differences (P>0.05) (Table-I).

**Table-I:** Comparison of VAS score and ODI values before surgery and during follow up.

*Time point*	*Average VAS score*	*Average ODI value*
Before surgery	8.11±0.37	83.40±6.20
24 h after surgery	2.82±0.46	28.50±4.80
1 month after surgery	2.47±0.42	25.40±6.30
3 month after surgery	2.43±0.46	26.50±6.40
6 month after surgery	3.02±0.38	35.80±7.50
12 month after surgery	3.13±0.66	36.40±4.70

### The dose of pain reliever after surgery

After surgery, nine patients stopped medication, 18 patients took half of the previous dose, and seven patients kept the previous dose. 79.4% of patients (27/34) reduced the dose of pain reliever or did not take pain reliever anymore.

### Complications

After surgery, six patients had fever, but recovered after expectant treatment; two patients felt uncomfortable in the right lower limbs, but also relieved after expectant treatment. Infection, bleeding and pulmonary embolism were not observed in all patients. Moreover, the second fracture of the surrounding vertebrae and diseased vertebrae were not observed.

## DISCUSSION

Spinal metastatic tumors have a high incidence and death rate among bone metastatic tumors. It may result in the pathological fracture of vertebrae and compression of spinal cord and nerve root, besides pains in waist and back. Therefore, the stability of spine is required to be improved in the treatment of spinal metastatic tumors, besides the relief of pains.^9^,^10^ Though there are many clinical therapies for spinal metastatic tumors, they all have defects.

Percutaneous vertebroplasty means infusing biomaterials such as bone cement into vertebrae using minimally invasive technology. It can improve the compressive strength of diseased vertebrae, avoid the collapse and malformation of vertebrae, relieve pains, and enhance body functions. As reported, the remission rate of the method was 75% to 94%.^11^ However, the pain relief mechanism of the therapy has not been thoroughly known. But there are several possible action mechanisms.^12^-^16^ The first one is heat effect and toxic effect. The toxic effect and heat produced during the polymerization of polymethyl methacrylate lead to the necrosis of pain nerve endings or tumor tissues in vertebrae. The second is mechanical effect. The evenly distributed bone cement can play a function of mechanical support, which prevents the occurrence of small activities of vertebrae and pains. The last one is vascular effect. The infusion of bone cement induces the necrosis of pain nerve endings or tumor tissues in vertebrae by blocking the supporting veins of vertebrae. In this study, the success rate of surgery was relatively high and the VAS score and ODI values after surgery were significantly lower than those before surgery, indicating percutaneous vertebroplasty could significantly and rapidly relieve pains, which was consistent with the findings of Wang HW et al.^11^

In clinic, bone cement leakage is a commonly seen complication in percutaneous vertebroplasty, i.e., bone cement may leak to paravertebral soft tissues, canalis vertebralis, intervertebral disks and basivertebral veins. But there are no obvious symptoms in most cases. The incidence of bone cement leakage is 30% to 87.5%.^17^ But the complications associated to bone cement leakage are usually not severe. Gargin found that, the incidence of bone cement leakage was 4% and the incidence of spinal cord compression was 0.5% in percutaneous vertebroplasty.^18^ Surgery should be performed if corresponding symptoms and vital signs appear when the leaked bone cement oppresses nerve root or spinal cord. In this study, 15 patients had bone destruction in vertebra posterior border. Percutaneous vertebroplasty is effective for patients with bone destruction in vertebra posterior border, but it has high risks and the surgery should be operated by experienced doctors. Other complications include pulmonary embolism, secondary fracture of surrounding vertebrae, local and systemic infection and temporary fever and pain. For patients with metastatic tumors in vertebrae, percutaneous vertebroplasty can timely relieve pain and enhance function, and the effects can last to the end of follow up. In the long run, the therapy is also safe.

### Limitation of the study

The incidence of complications was extremely low in this study, which might be correlated to the small sample size. The postoperative survival time analysis was not involved in the study. Therefore, it is difficult to evaluate the influence of percutaneous vertebroplasty on the prognosis of spinal metastasis tumor. Further studies with large sample size are needed.

## CONCLUSION

Percutaneous vertebroplasty can improve the strength and stability of vertebrae, prevent further collapse of vertebrae, and relieve pain in waist and back. Moreover, the heat effect and toxic effect produced by polymethyl methacrylate can help kill tumor cells. Most patients can tolerate the surgery because of the small trauma, significant relief effect and few complications; it is a safe, effective and minimally invasive surgical method. But percutaneous vertebroplasty also has limitations in controlling spiral tumors. It can be combined with ordinary radiotherapy, radiofrequency ablation and radioactive seeds implantation. Tumor is a systemic disease; therefore, primary tumors should also be treated to extend the survival time of patients.

### Authors’ Contribution

***YLL & LMW:*** Study design, data collection and analysis.

***YQW, LZ, RPS & LMW:*** Manuscript preparation, drafting and revising.

***YLL & HYT:*** Review and final approval of manuscript.

## References

[1] Koyfman SA, Djemil T, Burdick MJ, Woody N, Balagamwala EH, Reddy CA (2012). Marginal recurrence requiring salvage radiotherapy after stereotactic body radiotherapy for spinal metastases. Int J Radiat Oncol Biol Phys.

[2] Witham TF, Khavkin YA, Gallia GL, Wolinsky JP, Gokaslan ZL (2006). Surgery insight: current management of epidural spinal cord compression from metastatic spine disease. Nat Clin Pract Neurol.

[3] Bartels RH, van der Linden YM, van der Graaf WT (2008). Spinal extradural metastasis: review of current treatment options. CA Cancer J Clin.

[4] Liljenqvist U, Lerner T, Halm H, Buerger H, Gosheger G, Winkelmann W (2008). En bloc spondylectomy in malignant tumors of the spine. Eur Spine J.

[5] Tian QH, Sun XQ, Lu YY, Wang T, Wu CG, Li MH (2016). Percutaneous vertebroplasty for palliative treatment of painful osteoblastic spinal metastases: a single-center experience. J Vasc Interv Radiol.

[6] Roedel B, Clarençon F, Touraine S, Cormier E, Molet-Benhamou L, Le Jean L (2015). Has the percutaneous vertebroplasty a role to prevent progression or local recurrence in spinal metastases of breast cancer?. J Neuroradiol.

[7] Lieberman IH, Dudeney S, Reinhardt MK, Bell G (2001). Initial outcome and efficacy of “kyphoplasty” in the treatment of painful osteoporotic vertebral compression fractures. Spine Phila Pa.

[8] Hadjipavlou AG, Tzermiadianos MN, Katonis PG, Szpalski M (2005). Percutaneous vertebroplasty and balloon kyphoplasty for the treatment of osteoporotic vertebral compression fractures and osteolytic tumours. J Bone Joint Surg Br.

[9] Clarençon F, Di Maria F, Cormier E, Sourour NA, Enkaoua E, Sailhan F (2013). Onyx injection by direct puncture for the treatment of hypervascular spinal metastases close to the anterior spinal artery: Initial experience. J Neurosurg Spine.

[10] Zhu SX, Gao LL (2014). Study on percutaneous vertebroplasty for the treatment of spinal metastases. Nei Mongol J TCM.

[11] Wang HW, He SC, Teng GJ, Fang W, Guo JH, Deng G (2010). Percutaneous vertebroplasty for the treatment of spinal metastasis: evaluation of the therapeutic efficacy. J Interv Radiol.

[12] Bae JW, Gwak HS, Kim S, Joo J, Shin SH, Yoo H (2016). Percutaneous vertebroplasty for patients with metastatic compression fractures of the thoracolumbar spine: clinical and radiological factors affecting functional outcomes. Spine J.

[13] Xiao QP, Wu CG, Wang T, Tian QH, Chen YD, Li MH (2015). Clinical application of percutaneous vertebroplasty combined with ^125i seeds implantation in treating bone metastases. J Clin Radiol.

[14] Bohner M, Gasser B, Baroud G, Heini P (2003). Theoretical and experimental model to describe the injection of a polymethylmethacrylate cement into a porous structure. Biomaterials.

[15] Chou D, Wang VY, Gupta N (2009). Transpedicular corpectomy with posterior expandable cage placement for L1 burst fracture. J Clin Neurosci.

[16] Deng G, He SC, Teng GJ, Fang W, Guo JH, Zhu GY (2005). Percutaneous vertebroplasty for spinal malignant tumors: a report of 173 cases. J Interv Radiol.

[17] Zhang JJ, Wang YG, Han K, Tang L, Hu H, Wu C (2013). Percutaneous vertebroplasty combined with zoledronic acid for the treatment of painful osteolytic spinal metastases in patients with breast cancer. J Vasc Interv Radiol.

[18] Gaffin SR, Yuan HA, Reiley MA (2001). New technologies in spine: kyphoplasty and vertebroplasty for the treatment of painful osteoporotic compression fractures. Spine.

